# A randomized controlled trial evaluating combination detection of HIV in Malawian sexually transmitted infections clinics

**DOI:** 10.1002/jia2.25701

**Published:** 2021-04-30

**Authors:** Jane S Chen, Mitch Matoga, Brian W Pence, Kimberly A Powers, Courtney N Maierhofer, Edward Jere, Cecilia Massa, Shiraz Khan, Sarah E Rutstein, Sam Phiri, Mina C Hosseinipour, Myron S Cohen, Irving F Hoffman, William C Miller, Kathryn E Lancaster

**Affiliations:** ^1^ Department of Epidemiology Gillings School of Global Public Health University of North Carolina at Chapel Hill Chapel Hill NC USA; ^2^ UNC Project Malawi Lilongwe Malawi; ^3^ Institute for Global Health and Infectious Diseases University of North Carolina at Chapel Hill Chapel Hill NC USA; ^4^ Lighthouse Trust Lilongwe Malawi; ^5^ Division of Epidemiology College of Public Health The Ohio State University Columbus OH USA

**Keywords:** clinical trials, HIV epidemiology, sexually transmitted infections/diseases, Africa, HIV care continuum

## Abstract

**Introduction:**

HIV diagnosis is the necessary first step towards HIV care initiation, yet many persons living with HIV (PLWH) remain undiagnosed. Employing multiple HIV testing strategies in tandem could increase HIV detection and promote linkage to care. We aimed to assess an intervention to improve HIV detection within socio‐sexual networks of PLWH in two sexually transmitted infections (STI) clinics in Lilongwe, Malawi.

**Methods:**

We conducted a randomized controlled trial to evaluate an intervention combining acute HIV infection (AHI) screening, contract partner notification and social contact referral versus the Malawian standard of care: serial rapid serological HIV tests and passive partner referral. Enrolment occurred between 2015 and 2019. HIV‐seropositive persons (two positive rapid tests) were randomized to the trial arms and HIV‐seronegative (one negative rapid test) and ‐serodiscordant (one positive test followed by a negative confirmatory test) persons were screened for AHI with HIV RNA testing. Those found to have AHI were offered enrolment into the intervention arm. Our primary outcome of interest was the number of new HIV diagnoses made per index participant within participants’ sexual and social networks. We also calculated total persons, sexual partners and PLWH (including those previously diagnosed) referred per index participant.

**Results:**

A total of 1230 HIV‐seropositive persons were randomized to the control arm, and 561 to the intervention arm. Another 12,713 HIV‐seronegative or ‐serodiscordant persons underwent AHI screening, resulting in 136 AHI cases, of whom 94 enrolled into the intervention arm. The intervention increased the number of new HIV diagnoses made per index participant versus the control (ratio: 1.9; 95% confidence interval (CI): 1.2 to 3.1). The intervention also increased the numbers of persons (ratio: 2.5; 95% CI: 2.0 to 3.2), sexual partners (ratio: 1.7; 95% CI: 1.4 to 2.0) and PLWH (ratio: 2.3; 95% CI: 1.7 to 3.2) referred per index participant.

**Conclusions:**

Combining three distinct HIV testing and referral strategies increased the detection of previously undiagnosed HIV infections within the socio‐sexual networks of PLWH seeking STI care. Combination HIV detection strategies that leverage AHI screening and socio‐sexual contact networks offer a novel and efficacious approach to increasing HIV status awareness.

## 1 INTRODUCTION

HIV diagnosis is the essential first step for people living with HIV (PLWH) to access HIV care, which can improve health outcomes and reduce the potential for onward transmission [1, 2, 3]. Although the scale‐up of routine HIV testing in medical settings has improved HIV detection globally [4], an estimated 19% of the nearly 38 million PLWH around the world are unaware of their HIV‐positive status [5]. Improved HIV detection strategies are crucial for increasing HIV status awareness worldwide.

One key testing gap is unrecognized acute HIV infection (AHI), which is the period of heightened transmission risk prior to the development of detectable antibodies [6]. Testing for AHI with RNA‐ or antigen‐based approaches improves HIV detection by enabling earlier diagnosis than with the standard serological testing [7]. This approach has proven particularly efficient in populations with high HIV incidence, including sexually transmitted infections (STI) clinic patients [4, 7, 8]. Assisted partner notification (aPN) strategies, in which medical staff help PLWH refer sexual partners for HIV testing, are another efficient means of identifying PLWH unaware of their HIV infection [9, 10]. Asking PLWH to refer their social contacts for HIV testing can also facilitate HIV diagnosis and offer a simple and inexpensive approach to widen healthcare engagement [11].

Combination detection strategies built from these approaches may be advantageous in high‐burden settings like Malawi, where national HIV prevalence and incidence are estimated at 9% and 33,000 new HIV cases per year, respectively, and the standard of care for HIV testing is serial rapid serological testing and passive partner referral [12, 13]. We developed an intervention incorporating AHI screening, the aPN strategy of contract partner notification, and social contact referral, to increase HIV detection among STI clinic patients and members of their social and sexual (socio‐sexual) networks. We assessed intervention efficacy through a randomized controlled trial (RCT) in two STI clinics in Lilongwe, Malawi.

## 2 METHODS

### 2.1 Study setting and population

Patients seeking outpatient STI services at Bwaila District Hospital or Kamuzu Central Hospital (KCH) in Lilongwe, Malawi were recruited to enrol in the two‐arm RCT between June 2015 and May 2019. Bwaila Hospital is the largest public hospital in Lilongwe and KCH is the largest tertiary hospital. In January 2016, KCH transitioned to referral‐based care, closing its STI clinic. All subsequent trial enrolment occurred at the Bwaila STI Clinic.

Per Malawian standard of care, STI patients were tested for HIV at the beginning of their visit, prior to study enrolment, using serial rapid serological tests [13]. Each patient whose first rapid test was negative was classified as HIV‐seronegative. Each patient whose first rapid test was positive was given a confirmatory antibody test; those with a positive confirmatory test were classified as HIV‐seropositive and those with a negative confirmatory test were classified as HIV‐serodiscordant.

Informed consent was obtained from all participants. For eligible HIV‐seropositive persons, consent was obtained after routine HIV rapid testing and after study arm assignment to allow for procedure‐specific consent (see below) [14]. For eligible HIV‐seronegative or HIV‐serodiscordant persons, consent was obtained after HIV rapid testing but prior to collection of specimens for acute HIV screening.

### 2.2 Study design

**Figure 1:**
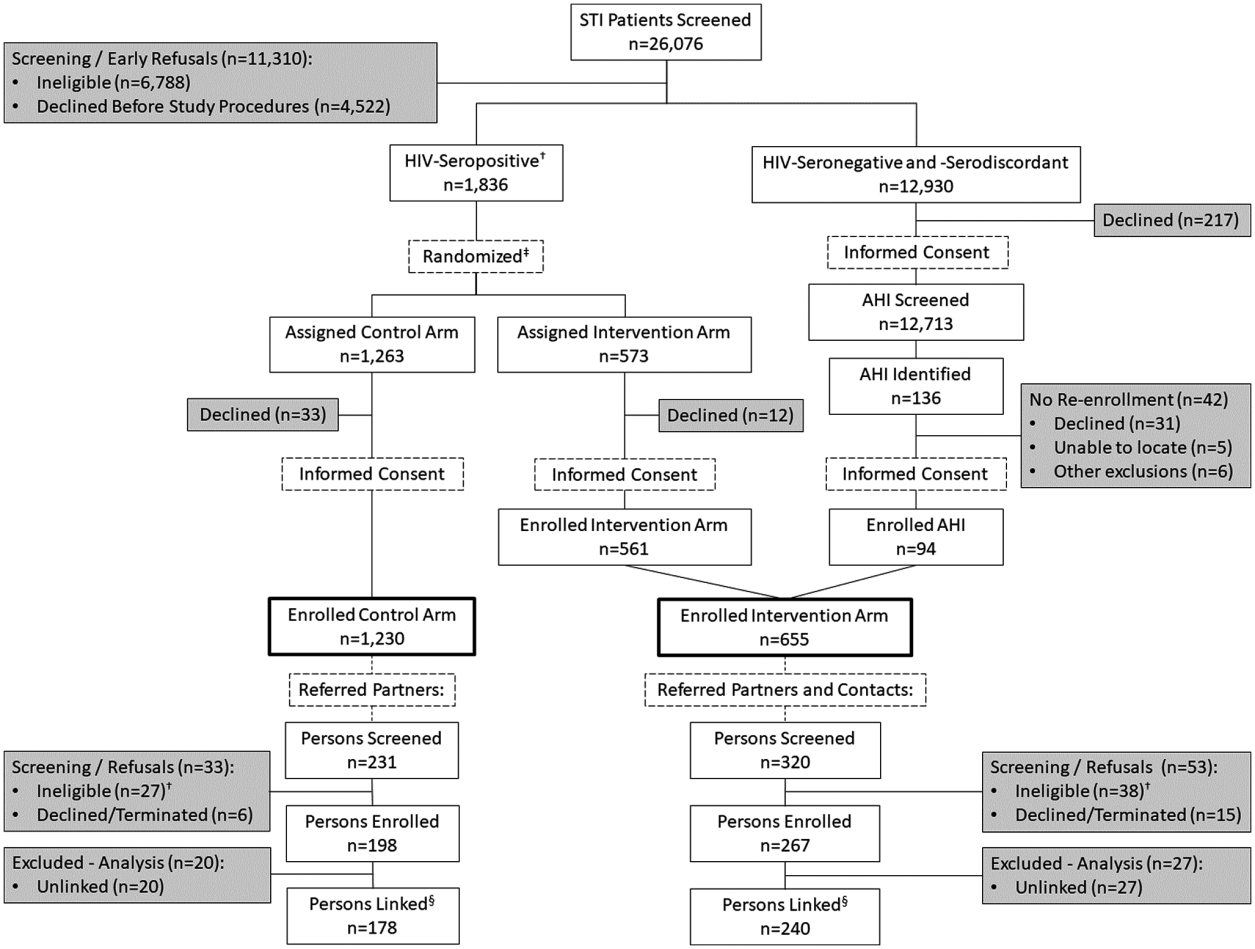
Trial screening and enrolment, 2015 to 2019. ^†^Prior to April 2017, persons previously diagnosed with HIV were ineligible for study enrolment. ^‡^Randomization started as 3:1 (control:intervention) and changed to 1:1 in April 2018. ^§^Linked to the original index participant and included in the analysis. AHI, acute HIV infection; STI, sexually transmitted infections.

After routine HIV status determination, potential participants were screened for study eligibility. STI patients were eligible for study participation if they were ≥18 years old, lived in Lilongwe District, and reported having sex in the previous six months. If eligible and newly HIV‐seropositive based on concordant positive rapid tests, participants were randomized to the standard of care (control) arm or the intervention arm (Figure 1). Following arm assignment, participants were given detailed information about the study and informed consent was obtained [14].

If eligible and HIV‐seronegative or ‐serodiscordant, participants were given detailed information about the study and consent was obtained. After consent, HIV‐seronegative or ‐serodiscordant participants received screening for AHI (see below). All participants with AHI were considered part of the intervention arm. Neither study staff nor participants were blinded.

Randomization at the start of the trial was 3:1 (control: intervention), and arm assignment was blocked in groups of 4 and 8 by study personnel uninvolved with participant enrolment. The target sample size was 1200 intervention arm participants and 3300 control arm participants. This ratio was selected to minimize disruption of clinical activities, and the sample size was expected to provide 87% power to detect an absolute difference of 0.04 between the intervention and control groups, assuming a control group referral proportion of 0.156. In response to recruitment reductions after the KCH study site closed, persons previously diagnosed with HIV became eligible for participation in April 2017, and the study randomization ratio switched to 1:1 in April 2018 to increase intervention arm enrolment specifically. Also, in April 2018, a travel reimbursement was implemented for all study participants. The reimbursement and its amount were determined by the local ethics committee.

### 2.3 Control arm

Control arm study procedures followed the Malawian standard of care. Participants were asked to passively refer up to five sexual partners from the previous six months to the STI clinic for HIV testing with participant‐specific referral cards (i.e. cards bearing the index participant’s ID code). Participants also completed a short behavioural questionnaire.

### 2.4 Intervention arm

Intervention arm procedures included contract partner notification, social contact referral and AHI screening, as described below. Participants provided a blood sample and completed an in‐depth behavioural survey.

Contract partner notification is a form of aPN where patients are encouraged to refer their sexual partners for testing but are also asked to provide partner contact information in case a partner does not return for testing within a certain period. In our study, intervention arm participants were asked to provide contact information for up to five sexual partners from the previous six months and were informed that clinic staff would attempt to contact partners not returning within seven days. Intervention arm participants were also given participant‐specific cards for referral. No index participant identifiers or clinical information was shared with partners.

For social contact referral, participants in the intervention arm were asked to refer up to five acquaintances who might benefit from HIV/STI services. Names or pseudonyms were used to help participants identify specific persons, but no contact information was collected. Participants were given participant‐specific referral cards to give to these social contacts. Referral type (sexual partner vs. social contact) was distinguishable by card colour.

For AHI screening, HIV‐seronegative and ‐serodiscordant participants provided a blood sample for HIV RNA detection with Abbott RealTime HIV‐1 PCR assays in pooled groups [7]. Persons who were seronegative or serodiscordant with HIV RNA ≥5000 copies/ml were considered to have AHI. Study staff initiated contact with all persons with AHI within one day of a positive AHI result. Contacted persons were referred to HIV care and offered enrolment into the intervention arm.

### 2.5 Referred persons

All persons who presented to the STI clinic with a sexual or social referral card had their HIV serostatus determined per clinical standard of care. Study eligibility was assessed and enrolment was offered, with subsequent study procedures depending on the arm of the original index participant. Specifically, in the control arm, enrolled HIV‐seropositive partners were encouraged to refer their own partners (i.e. passive partner referral), and enrolled HIV‐seronegative and ‐serodiscordant partners received no further testing. In the intervention arm, enrolled HIV‐seropositive partners/contacts received contract partner notification and social contact referral procedures, and enrolled HIV‐seronegative and ‐serodiscordant partners/contacts underwent AHI screening. All referred and enrolled sexual partners and social contacts, including those who were partners/contacts of partners/contacts, were linked back to the original HIV‐seropositive or acutely infected index participant. If a person reported that they or the person who referred them had lost their referral card, the index participant was able to be looked up by name in the study records. Due to changing enrolment criteria, referred persons previously diagnosed with HIV were not enrolled at the start of the trial and were therefore not counted toward outcomes in either arm until April 2017. Systems were created to document any social harms reported during study procedures, though none were reported.

### 2.6 Study outcomes

Because the study aimed to improve HIV testing within the socio‐sexual networks of PLWH seeking STI care, all outcomes in this analysis were based on referred sexual partners and social contacts. Furthermore, all outcomes in this analysis were assessed among referred persons who enrolled in the study and were able to be linked back to the original HIV‐seropositive or acutely infected index participant, excluding sexual partners and social contacts who were ineligible for study participation (due to age, prior HIV diagnosis before inclusion criteria changed, etc.) or unlinked to their referring index participant (e.g. computer error recording IDs).

Our primary outcome of interest was the number of referred persons receiving a new HIV diagnosis per index participant. Per study protocol, new HIV diagnoses were determined differently by arm: by serological tests in the control arm and by both serological and PCR tests in the intervention arm. We assessed three other outcomes of interest: referred persons irrespective of HIV status, referred sexual partners and referred PLWH (i.e. including those previously diagnosed with HIV). For each outcome, we examined three metrics: the number referred per index participant, the proportion of index participants with ≥1 referral and the number of index participants needed to receive the intervention (NNI) to result in one referral.

### 2.7 Statistical analysis

To evaluate the intervention on the basis of “number referred per index participant,” we used negative binomial regression or Poisson regression with a scaled deviance when a negative binomial model would not converge [15]. To test for differences between arms in proportions referring ≥1 person of a given outcome type, we used Fisher’s Exact Test. Finally, we calculated the number of index participants needed to receive the intervention (NNI) to refer one person of a given type, calculated as: $\frac{1}{{\left(\#\text{referred/}\#\text{index}\right)}_{intervention}‐{\left(\#\text{referred}/\#\text{index}\right)}_{control}}$ rounded up to the nearest integer.

We conducted several sensitivity analyses to address specific aspects of the study design, each time reassessing our main outcome of new HIV diagnoses made per index participant. To account for any unintended confounding during arm assignment, we repeated our main analysis adjusting for the sex, marital status and new versus previous HIV diagnosis of the index participant. To account for the inclusion of index participants with AHI in the intervention arm only, we repeated our main analysis restricting to HIV‐seropositive participants. To understand the impacts of including previously diagnosed persons midway through the trial, we repeated our main analysis with the addition of an interaction term for new (vs. previous) HIV diagnosis. Finally, to assess the impacts of the travel reimbursement, we repeated the main analysis with an interaction term for receiving (vs. not receiving) the reimbursement. Interactions were considered significant at α = 0.05.

Analyses were conducted using SAS v9.4 (Cary, NC, USA). All study procedures received ethical approval from the University of North Carolina Institutional Review Board and the Malawian National Health Services Research Committee. The RCT is registered at clinicaltrials.gov (NCT number: NCT02467439).

## 3 RESULTS

Between June 2015 and May 2019, 26,076 STI clinic patients were screened for study enrolment (Figure 1). Of those, 11,310 (43%) were ineligible or declined/terminated study participation (26% and 17% respectively). The most common reasons for ineligibility were reporting no sex in the previous six months (42%), living outside Lilongwe (27%) and being <18 years old (23%). The most common reason for refusing participation was having no time (55%).

In total, 14,504 index participants enrolled (56% of screened participants; 75% of eligible participants). Of those, 1230 (8%) were HIV‐seropositive participants randomized into the control arm, and 561 (4%) were HIV‐seropositive participants randomized into the intervention arm. An additional 12,713 (88%) were HIV‐seronegative or ‐serodiscordant participants who were screened for AHI. Within the latter group, 136 participants (1%) were diagnosed with AHI, 94 (69%) of whom enrolled into the intervention arm. This resulted in 1885 index participants, with 1230 in the control arm and 655 (561 seropositive, 94 AHI) in the intervention arm.

### 3.1 Index participants

Most randomized HIV‐seropositive index participants were newly diagnosed with HIV (control: 71%; intervention: 65%), female (control: 60%; intervention: 62%) and married (control: 68%; intervention: 62%) (Table 1). Approximately half were 25 to 34 years old (control: 47%, intervention: 49%), with approximately one‐quarter older and one‐quarter younger.

**Table 1:** Demographic and behavioural characteristics of index participants by study arm

Characteristic	Control – Index	Intervention – Index		
	All	All	Seropositive	Acute
Total	1230	655	561	94
	n (%)	n (%)	n (%)	n (%)
HIV status				
Previously HIV‐positive, ART user	306 (25)	165 (25)	165 (29)	0 (0)
Previously HIV‐positive, no ART	50 (4)	31 (5)	31 (6)	0 (0)
New HIV‐seropositive diagnosis	874 (71)	365 (56)	365 (65)	0 (0)
Acute HIV diagnosis	0 (0)	94 (14)	0 (0)	94 (100)
Sex				
Male	490 (40)	257 (39)	213 (38)	44 (47)
Female	725 (60)	395 (61)	345 (62)	50 (53)
Missing	15	3	3	0
Age				
18 to 24	305 (25)	175 (27)	137 (25)	38 (40)
25 to 34	571 (47)	303 (47)	265 (49)	38 (40)
35 to 44	281 (23)	138 (22)	123 (23)	15 (16)
≥45	58 (5)	24 (4)	21 (4)	3 (3)
Missing	15	15	15	0
Marital status				
Never married	152 (12)	93 (14)	72 (13)	21 (23)
Married	828 (68)	388 (60)	344 (62)	44 (48)
Divorced/widowed	237 (19)	166 (26)	140 (25)	26 (29)
Missing	13	8	5	3

	Median (IQR)	Median (IQR)	Median (IQR)	Median (IQR)
Behavioural characteristics				
Number partners in past 4 weeks	1 (1, 1)	1 (1, 1)	1 (1, 1)	1 (1, 1)
Number sex acts in past 4 weeks	4 (2, 12)	5 (2, 10)	5 (2, 12)	4 (1, 8)

ART, antiretroviral therapy; IQR, interquartile range.

Within the intervention arm, index participants with AHI had a more even sex distribution (53% female vs. 62%), were slightly younger (40% were 18 to 24 years old vs. 25%), and were more likely to be never married (23% vs. 13%) compared with HIV‐seropositive index participants.

### 3.2 Referred persons

Across all referral chains, 231 sexual partners were referred to the STI clinic by control arm participants, and 320 sexual partners and social contacts were referred by intervention arm participants (Figure 1). Of these, 198 (86%) partners in the control arm, and 267 (83%) persons in the intervention arm were eligible and enrolled in the study. Among referred persons who did not enrol, more than half (65%) were ineligible due to having a known HIV diagnosis before study eligibility changed to include them. Of the 198 and 267 enrolled participants in the control arm and intervention arms, respectively, 178 (90%) and 240 (90%) were able to be linked to the original index participant. The longest referral chain was four degrees of separation, though the most common referral chain was one referred person.

Per protocol, all 178 linked persons in the control arm were sexual partners. In the intervention arm, 157 (66%) of the linked referrals were sexual partners, 81 (34%) were social contacts and 2 were unrecorded (Table 2). Among sexual partners, 53% were men in the control arm and 63% were men in the intervention arm. The majority were married (control: 83%; intervention: 85%) and about half were 25 to 34 years old (control: 46%; intervention: 49%). About half of the sexual partners in both arms tested HIV‐negative (control: 54% HIV‐seronegative per serological testing; intervention: 51% HIV‐negative per serological and PCR testing), and approximately a quarter reported being previously HIV‐diagnosed in both arms (control: 25%; intervention: 29%). The majority of those previously HIV‐diagnosed reported taking antiretroviral therapy (ART) in both arms (control: 95%; intervention: 87%).

**Table 2:** Demographic and behavioural characteristics of referred, enrolled and linked persons, by study arm

Characteristic	Control arm: total	Intervention arm: total	Intervention arm: sexual partners^a^	Intervention arm: social contacts^a^
Total	178	240	157	81
	n (%)	n (%)	n (%)	n (%)
Referral type				
Sexual partner	178 (100)	157 (66)	157 (100)	0 (0)
Social contact	0 (0)	81 (34)	0 (0)	81 (100)
Missing	0	2	0	0
HIV status^b^				
HIV seronegative/serodiscordant	96 (54)	–	–	–
HIV negative	–	138 (58)	80 (51)	57 (70)
Acute HIV	–	5 (2)	5 (3)	0 (0)
New HIV‐seropositive diagnosis	38 (21)	33 (14)	26 (17)	6 (7)
Previously HIV‐positive, ART user	42 (24)	55 (23)	40 (25)	15 (19)
Previously HIV‐positive, no ART	2 (1)	9 (4)	6 (4)	3 (4)
Sex				
Male	93 (53)	133 (56)	97 (63)	34 (43)
Female	84 (47)	104 (44)	58 (37)	46 (58)
Missing	1	3	2	1
Age				
18 to 24	51 (29)	52 (23)	30 (20)	20 (26)
25 to 34	82 (46)	107 (47)	73 (49)	34 (44)
35 to 44	36 (20)	57 (25)	40 (27)	17 (22)
≥45	8 (5)	14 (6)	7 (5)	7 (9)
Missing	1	10	7	3
Marital status				
Never married	22 (13)	25 (10)	13 (8)	10 (12)
Married	146 (83)	189 (79)	133 (85)	56 (69)
Divorced/widowed	7 (4)	25 (10)	10 (6)	15 (19)
Missing	3	1	1	0

	Median (IQR)	Median (IQR)	Median (IQR)	Median (IQR)
Behavioural characteristics				
Number partners in past 4 weeks	1 (1, 1)	1 (1, 1)	1 (1, 1)	1 (1, 2)
Number sex acts in past 4 weeks	5 (2, 12)	5 (1, 9)	5 (2, 10)	5 (1, 8)

^a^ Two referred persons were unable to be classified as a sexual partner or a social contact

^b^ HIV testing protocols in each arm resulted in different classification schemes across arm. In the control arm, persons were only tested with serial rapid tests, per standard of care, resulting in serological diagnoses only. In the intervention arm, HIV‐seronegative and HIV‐serodiscordant participants were further screened for AHI, resulting in virological diagnoses. ART, antiretroviral therapy; IQR, interquartile range

Among persons referred in the intervention arm, a greater percentage of social versus sexual contacts were HIV‐negative (70% vs. 51%) and women (58% vs. 37%). Fewer social contacts were newly diagnosed with HIV (7% vs. 20%) and married (69% vs. 85%) compared with sexual contacts.

Thirty‐eight referred persons in each arm were newly diagnosed with HIV through their study participation (control: 38 from 1230 index participants; intervention: 38 from 655 index participants), including five sexual partners in the intervention arm who were newly diagnosed with AHI (3% of referred persons screened for AHI).

### 3.3 Efficacy analyses

The intervention was efficacious as measured by our primary outcome, with 1.9 times (95% CI: 1.2 to 3.1) as many referred persons newly diagnosed with HIV per index participant in the intervention versus control arm (0.06 vs. 0.03 referred persons with a new HIV diagnosis per index participant in the intervention vs. control arm; Table 3). The intervention was similarly efficacious in terms of total persons, sexual partners and PLWH referred per index, with ratios comparing the intervention to control arm of 2.5 (95% CI: 2.0 to 3.2), 1.7 (95% CI: 1.4 to 2.0) and 2.3 (95% CI: 1.7 to 3.2) respectively.

**Table 3:** Count, proportion and NNI outcomes

Outcome	N	Number referred per index		Proportion referred ≥1		NNI
		Count (95% CI)	Ratio (95% CI)	%	Ratio (95% CI)	
Referred persons who received a new HIV diagnosis						
Control arm	38	0.03 (0.02, 0.04)		3.0%	–	–
Intervention arm	38	0.06 (0.04, 0.08)	1.9 (1.2, 3.1)	4.9%	1.6 (1.0, 2.6)	37
Total persons referred						
Control arm	178	0.14 (0.12, 0.17)	–	12.9%	–	–
Intervention arm	240	0.37 (0.31, 0.43)	2.5 (2.0, 3.2)	24.9%	1.9 (1.6, 2.3)^a^	5
Sexual partners referred						
Control arm	178	0.14 (0.13, 0.16)	–	12.9%	–	–
Intervention arm	157	0.24 (0.21, 0.27)	1.7 (1.4, 2.0)	22.3%	1.7 (1.4, 2.1)^a^	11
Persons living with HIV referred						
Control arm	82	0.07 (0.05, 0.08)	–	6.3%	–	–
Intervention arm	102	0.16 (0.12, 0.19)	2.3 (1.7, 3.2)	12.4%	2.0 (1.5, 2.6)^a^	12

NNI, number needed to receive the intervention.

^a^ Statistically significant (α = 0.05) with Fisher’s Exact Test.

After adjusting for the index participant’s sex, HIV diagnosis status and marital status, we estimated a similar ratio of 2.0 (95% CI: 1.2 to 3.2) comparing the number of referred persons newly diagnosed with HIV per index participant in the intervention versus control arm (Table 4). When we restricted to HIV‐seropositive participants (i.e. excluding participants with AHI), we estimated a smaller ratio of 1.6 (95% CI: 0.9 to 2.6).

**Table 4:** Sensitivity analyses of the main outcome (new HIV diagnoses per index participant)

Sensitivity analysis	Index (N)	New HIV diagnoses per index (95% CI)	Ratio (95% CI)	Interaction term p value
Adjusted model^a^				
Control arm	1202	0.03 (0.02, 0.06)	–	–
Intervention arm	644	0.07 (0.03, 0.13)	2.0 (1.2, 3.2)	
Restricted to HIV‐seropositive participants^b^				
Control arm	1230	0.03 (0.02, 0.04)	–	–
Intervention arm	561	0.05 (0.03, 0.07)	1.6 (0.9, 2.6)	
Differential effects according to index diagnosis status^c^				
Previous positive				0.23
Control arm	356	0.02 (0.01, 0.04)	–	
Intervention arm	196	0.06 (0.03, 0.11)	3.3 (1.2, 9.5)	
New positive				
Control arm	874	0.04 (0.03, 0.05)	–	
Intervention arm	459	0.06 (0.04, 0.09)	1.6 (0.9, 2.8)	

^a^ Adjusted for sex, new or previous HIV diagnosis, marital status of the index participant

^b^ acute participants and their referrals excluded

^c^ interaction term for new versus previous HIV diagnosis

Although not statistically significant, we found that the intervention had differential effects among index participants with new versus previous HIV diagnoses (*p* = 0.23). Specifically, previously HIV‐diagnosed participants in the intervention arm referred 3.3 times as many persons receiving an HIV diagnosis as in the control arm, whereas newly diagnosed participants in the intervention arm referred only 1.6 times as many persons receiving an HIV diagnosis as those in the control arm. Our effect estimates and confidence intervals for the intervention versus control before and after the travel reimbursement were very similar (Table S1).

In total, 4.9% of index participants in the intervention arm referred ≥1 person unaware of their HIV‐positive status, compared to 3.0% in the control arm (ratio: 1.6; 95% CI: 1.0 to 2.6) (Table 3). Participants receiving the intervention were also more likely than their control‐arm counterparts to refer ≥1 person of any HIV status (ratio: 1.9; 95% CI: 1.6 to 2.3), ≥1 sexual partner (ratio: 1.7; 95% CI: 1.4 to 2.1) and ≥1 PLWH (ratio: 2.0; 95% CI: 1.5 to 2.6).

In expectation, 37 people would need to receive the intervention for one additional HIV case to be detected relative to standard of care conditions. Five would need to receive the intervention to refer an additional person, 11 to refer an additional sexual partner, and 12 to refer an additional PLWH (Table 3).

## 4 DISCUSSION

We assessed the effect of an intervention incorporating contract partner notification, social contact referral and AHI screening on HIV detection within the socio‐sexual networks of PLWH seeking STI care in Lilongwe, Malawi. This combination detection intervention increased all referral outcomes of interest relative to the standard of care, including our primary outcome of new HIV diagnoses made.

As an efficient means of identifying new HIV diagnoses within socio‐sexual networks, combining AHI screening with aPN in STI clinics has the potential for substantial public health impact [7]. Testing sexual partners of persons with AHI reaches people who are either at high risk of HIV acquisition or who may have recently transmitted HIV, a critical population to test for HIV [16]. Testing social contacts and sexual partners of PLWH through AHI screening decreases the diagnostic window, allowing for earlier identification of infection relative to rapid antibody testing [17].

Assisted partner notification (aPN) as a standalone service has been recommended by the World Health Organization (WHO) since 2016 [10, 18]. We built upon the strategy and assessed how additional services could improve HIV detection. Despite these additions, however, the overall referral rates were lower than expected [19] resulting in a larger NNI for new HIV diagnoses than was seen in other aPN studies in Kenya [20], Mozambique [21] and Cameroon [22, 23]. Due to constraints of the existing health care system in Malawi, index participants in our study were not contacted after their clinic visit, and HIV testing by partners and social contacts was only captured when performed at our study sites. In Kenya, Cameroon and Mozambique, index participants had follow‐up visits, and HIV testing could occur in non‐study locations, including the home in Kenya and Cameroon. Such differences likely resulted in increased partner testing through greater study investment among index participants and easier testing logistics for their partners [19]. Our trial may represent a more real‐world version of an aPN programme implemented in a busy clinical setting with transient participant interaction. As such, additional analyses to better understand the limits of the intervention are needed. Gender, marital status and the HIV status of potential referrals likely impact the behaviour of both index participants and their referrals and understanding structural and social barriers to referral and participation are important areas of future research.

Sixteen percent of new diagnoses made through the intervention were among social contacts. Though there may have been misclassification about referral type, social contact referral remains an inexpensive way to increase HIV testing. For those who are not comfortable naming sexual partners, social contact referral offers a mechanism for PLWH to refer persons without needing to disclose sexual relationships. This benefit is especially relevant given that 11% of our screened participants reported no sex in the past six months despite seeking STI care.

In our study, screening for AHI among STI clinic patients identified 136 new cases of HIV (1% of screened participants), and five AHI cases were identified among referred partners (3% of screened referrals). When we excluded participants with AHI from our analysis, intervention efficacy decreased, underscoring the importance of AHI screening in the intervention. We note that because we focused on new diagnosis arising from referrals, we did not consider the number of index participants diagnosed with AHI in our outcomes, therefore underestimating the full impact of the intervention. Although PCR testing strategies to detect AHI may be cost‐prohibitive in some settings, fourth‐generation antigen‐antibody tests may mitigate costs while still reducing the HIV window period relative to rapid tests [24].

About 25% of eligible participants declined enrolment, reducing our planned sample size and potentially biasing our estimates and limiting the generalizability of our findings. A high proportion of persons who declined study participation cited a lack of time, likely due to the time required for informed consent and study procedures. Should the intervention be implemented as a future standard of care, these processes would be eliminated; however, persons still might opt‐out of procedures to shorten their clinic visits, decreasing the intervention’s reach. While we found the intervention to be successful, barriers to widespread implementation remain. The necessary investment in personnel, patient tracking systems and laboratory facilities is significant. Cost‐effectiveness analyses would be informative for implementation [25, 26, 27, 28, 29].

A unique component of our study was the ultimate inclusion of previously diagnosed PLWH, as other aPN studies tend to restrict to intervening among persons newly diagnosed with HIV [20, 23]. Interestingly, we found that the intervention may have had a stronger effect among previously diagnosed index participants versus those newly diagnosed. Participants newly diagnosed with HIV may feel a greater sense of urgency when passively referring partners, resulting in a higher level of referral through the standard of care. Meanwhile, those previously diagnosed may have already discussed their HIV status with their partners, and therefore may be less compelled to refer them passively, causing a lower level of referral in the control arm and thus a greater potential for an intervention effect.

## 5 CONCLUSIONS

Our intervention combining AHI screening, contract partner notification and social contact referral in STI clinics in Malawi was efficacious in bringing persons unaware of their HIV‐positive status into the STI clinic for HIV testing. Our findings support the current WHO guidelines on aPN, but suggest that in busy clinic settings, referral rates may be lower than anticipated. As the success of HIV treatment as prevention [2] and other HIV prevention strategies [30] shifts the HIV epidemic into vulnerable sub‐populations [12, 31], novel solutions for engaging difficult‐to‐reach persons unaware of their HIV status will become increasingly vital. Combination detection can improve HIV detection beyond the status quo.

## Competing Interests

MSC has acted in an advisory role for Merck and Gilead. The authors declare no other potential competing interests.

## Authors’ contributions

WCM, KEL, SP, KAP and SER contributed to study design. EJ, CM and MM contributed to data collection. EJ, CNM and JSC contributed to data analysis. WCM, KEL, IFH, BWP, KAP, CNM and JSC contributed to data interpretation. MM, BWP, KAP, CNM, EJ, CM, SK, SER, SP, MCH, MSC, IFH, WCM, KEL and JSC contributed to manuscript preparation.

## Abbreviations

AHI, acute HIV infection; aPN, assisted partner notification; ART, antiretroviral therapy; KCH, Kamuzu Central Hospital; NNI, number needed to receive the intervention; PCR, polymerase chain reaction; PLWH, Person(s) living with HIV; RCT, randomized controlled trial; STI, sexually transmitted infection; WHO, World Health Organization.

## Supporting information


**Table S1**. Sensitivity analysis of the main outcome (new HIV diagnoses per index participant) and the travel reimbursement
