# Supplementary material for: A randomized controlled trial evaluating combination detection of HIV in Malawian sexually transmitted infections clinics
Source: J Int AIDS Soc. 2021 Apr 30;24(4):e25701. doi: 10.1002/jia2.25701 (PMC8085969; doi:10.1002/jia2.25701)
Supplement: Supplementary file 1 — Table S1. Sensitivity analysis of the main outcome (new HIV diagnoses per index participant) and the travel reimbursement [file JIA2-24-e25701-s001.docx]

Supplemental Table 1. Sensitivity analysis of the main outcome (new HIV diagnoses per index participant) and the travel reimbursement

|  | Index  (N) | New HIV Diagnoses Per Index (95 CI) | Ratio (95% CI) | Interaction Term  P Value |
| --- | --- | --- | --- | --- |
| **Without Travel Reimbursement** |  |  |  | 0.57 |
| Control Arm | 1,045 | 0.03 (0.03, 0.04) | -- |  |
| Intervention Arm | 430 | 0.05 (0.04, 0.07) | 1.7 (1.3, 2.3) |  |
| **With Travel Reimbursement** |  |  |  | -- |
| Control Arm | 185 | 0.03 (0.02, 0.05) | -- |  |
| Intervention Arm | 225 | 0.07 (0.05, 0.09) | 2.1 (1.3, 3.4) |  |
